# MWCNT/Ruthenium hydroxide aerogel supercapacitor production and investigation of electrochemical performances

**DOI:** 10.1038/s41598-022-17286-w

**Published:** 2022-07-27

**Authors:** Satiye Korkmaz, İshak Afşin Kariper, Ceren Karaman, Onur Karaman

**Affiliations:** 1 Department of Electrical-Electronics Engineering, Faculty of Engineering, Karabuk University, 78050 Karabük, Turkey; 2 Education Faculty, Erciyes University, 38039 Kayseri, Turkey; 3 Department of Electricity and Energy, Akdeniz University, 07058 Antalya, Turkey; 4 Department of Medical Imaging Techniques, Akdeniz University, 07058 Antalya, Turkey

**Keywords:** Chemistry, Energy science and technology, Materials science, Physics

## Abstract

In this study, the material obtained from the sonication of the double-walled carbon nanotube and ruthenium chloride was produced as an aerogel. Then, symmetrical supercapacitor devices were made using them, and their electrochemical properties were investigated. XRD and FTIR were used in the structural analysis of the aerogel, STEM in surface images, and elemental analyses in EDX. Electrochemical analysis was performed by galvanostat/potentiostat. From the cyclic voltammetry analysis, the highest specific capacitance for MWCNT/Ruthenium hydroxide aerogels was achieved as 423 F/g at 5 mV/s. On the other hand, the corresponding values calculated from the charge–discharge curves were found to be 420.3 F/g and 319.9 F/g at the current densities of 0.5 A/g and 10.0 A/g, respectively. The capacitance retention of as-synthesized aerogel was 96.38% at the end of the 5000 consecutive consecutive cyclic voltammetry cycles.

## Introduction

Today, the energy need arising from the increasing population and industrialization cannot be met due to limited resources; consequently, the gap between energy production and consumption is rapidly growing. In this case, the use of available energy resources more effectively becomes increasingly important. The efficient storage of the energy obtained from renewable energy sources and the development of the most appropriate transformations will help meet the rapid increase in energy demand. In this context, supercapacitors (SCs) offer various advantages, including high power density, fast charge–discharge capability, and long cycle life. Therefore, they have garnered substantial attention as to be employed as the supreme energy storage systems in broad application fields^1–5^. However, since supercapacitors’ low energy density will limit their future applications, the engineering of high-performance SCs has scientific and industrial importance^6^. In recent studies, researchers have focused on different transition metal oxides (TMO), which have very good electrochemical performance and environmentally friendly properties, to be used in SCs. Metal oxides, in general, have a substantially greater energy density than typical carbonaceous structures and are more electrochemically stable than polymeric materials^7^. RuO_2_ is the first transition metal oxide reported for SCs. Its unique properties such as high specific capacitance (up to 1580 F/g), stability, large faradaic activity, ion adsorption, and good electrical conductivity made it one of the most notable candidates to be used in SC^8–10^.

CNTs, on the other hand, have excellent electrical conductivity, which, because of the unique physical architecture of the covalent sp2 bonding between carbon atoms, effectively lowers the resistivity of the system. They can serve as agents by developing a conductive network to obtain high-performance electrodes owing to their outstanding mechanical properties. CNTs assist in establishing a large electrolyte–electrode interface thanks to the high specific surface area (1300 m^2^g^−1^) and fulfill successful energy storage, in addition to their chemical stability^11–20^. However, CNTs cannot be used directly as active material; therefore, they should be combined with some metal oxides (RuO_2_, MoO_2_, etc.) or conductive polymers to achieve a higher energy density. On the other hand, aerogels having low density (0.003–0.15 kg/m^3^), high porosity, and large surface areas (500–1000 m^2^/g) are known as the lightest solid materials. Because of their tunable pore sizes and surface areas, as well as their mechanical strength and peculiar physicochemical properties, aerogels have a bright future for supercapacitor applications. Along with their three-dimensional network structure, carbon aerogels have excellent electrical conductivity.CNT aerogels are a good alternative as supercapacitor electrode material since they offer a large specific surface area, electrical conductivity, lightweight nature, and high mechanical strength^21^. Electrochemical performances of nanocomposites have recently been tested under various types of electrolytes.

RuO_2_-based composites containing "half-graphene" were obtained from the longitudinal division of CNTs in different aqueous media, such as 1 M KOH, 1 M H_2_SO_4_, and 1 M Na_2_SO_4_, by adding 40.0% metal oxide; their specific capacitances were found to be 453.7 Fg^−1^, 415.7 F/g, and 287.5 F/g, respectively^22^. Hydrous-RuO_2_ nanoparticles were uniformly coated on multi-walled carbon nanotubes (MWCNT) using the hydrothermal technique; they exhibited a gravimetric capacitance of 1585 Fg^−1^ at a voltage scan rate of 2 mVs^−1^ in the 0–1.2 V operating potential range^23^. A few years ago, Das et al. deposited RuO_2_ on specially designed porous SWCNT films by electrodeposition. They reported 1715 Fg^−1^ gravimetric capacitance for RuO_2_-based energy storage devices^24^. Asim et al. achieved a high specific capacitance of 176 Fg^−1^ at 2 mA cm^2^ for a hybrid nanocomposite consisting of CNT-decorated RuO_2_nanorods (NRs)^25^. Arabale et al. obtained a specific capacitance of 80 F.g^−1^ from multi-walled carbon nanotubes (MWCNTs) functionalized with aqueous RuO_2_ in 1 M sulfuric acid. This figure is significantly higher than unfunctionalized MWCNTs (30 F/g) in the same environment^26^. Liu revealed that micro-supercapacitors with hydrated RuO_2_ interdigital electrodes exhibit a specific capacitance of 10.5 mF/cm at 0.05 V/s^27^. Arnold obtained a specific capacitance of 720 F/g for high-capacity aqueous ruthenium oxide micro ultracapacitors. Sugimoto synthesized mesoporous RuOx electrodes by electrodeposition and achieved a specific capacitance of 400 F/g^28,29^. In this study, CNT/RuO_2_ aerogels were synthesized to be used as supercapacitor electrodes, and their electrochemical performances were investigated in 3.0 M H_2_SO_4_ liquid electrolyte. Herein, it was aimed to fabricate a hybrid electrode material to be employed in high-performance supercapacitor cells. In this regard, MWCNT/Ruthenium hydroxide aerogels were fabricated via a facile fabrication pathway, and their electrochemical performances as supercapacitor electrode material were examined in 3.0 M H_2_SO_4_ liquid electrolyte system. On the other hand, the physicochemical characterizations and elemental compositions of as-synthesized nanohybrid aerogel were conducted via SEM–EDX, XRD, and FTIR analysis. The cyclic voltammetry (CV) and galvanostatic charge–discharge (GCD) analysis were implemented the investigate the electrochemical performance of as-assembled supercapacitor cells. To the best of the authors’ knowledge, this is the first work reported on MWCNT/Ruthenium hydroxide aerogel electrodes-based supercapacitors; thus, it can be speculated that this work may contribute to the literature and guide research on the subject.

## Method

### MWCNT/Ruthenium hydroxide aerogel synthesis

2.0 mg.mL^−1^ of MWCNT and 0.5 mg.mL^−1^ of RuCl_3_ aqueous dispersions were mixed with a volumetric ratio of 5:6 to prepare the MWCNT/Ruthenium hydroxide aqueous dispersion, followed by 20 ml of the as-obtained dispersion were frozen at −10° C. Afterward, the dispersion at −10° C was treated at −78° C under vacuum in a lyophilizer overnight. Finally, the as-obtained sample was labeled as MWCNT/Ruthenium hydroxide aerogel.

1 $$ {\text{RuCl}}_{{3}} + {\text{3H}}_{{2}} {\text{O}} \to {\text{Ru}}\left( {{\text{OH}}} \right)_{{3}} + {\text{3HCl}} $$

### Electrocanalytical investigations

Certain amounts of MWCNT/Ruthenium hydroxide aerogel as active material (85% wt.), carbon black as a conductive additive (10% wt.), and polyvinylidene fluoride as a binder (5% wt.) were homogeneously dispersed in N-methyl-2-pyrrolidone (NMP) via ultrasonication over 30 min. A disk current collector (d = 1.51 cm) coated with the electrode slurry by casting method, was dried in a vacuum oven overnight at 80 °C to eliminate the probable NMP contamination. The 3.0 M H_2_SO_4_ electrolyte soaked electrodes and separator were used to assemble the symmetrical supercapacitor cells. For this purpose, two identical MWCNT/Ruthenium hydroxide aerogel-based electrodes were replaced in the anode and cathode sides of the CR2032 coin cell, they were separated by a cellulose film separator, followed by sealed by a hydraulic crimper. The total mass loading on each electrode was set to 2.5 mg cm^−2^.

The IviumStat potentiostat/galvanostat was employed to characterize the electrochemical properties of the symmetric supercapacitor. The electrochemical behaviors of as-assembled supercapacitor cells were examined in a potential scan rate range of 5 to 100 mV s^−1^ via cyclic voltammetry investigations, as well as the galvanostatic charge/discharge analysis acquired at the current density range of 0.5 to 10 A g^−1^ and operating voltage range of 0–1.0 V. 5000 consecutive CV cycles were recorded at a potential scan rate of 100 mV s^−1^ to assess the cycling stability of electrode material.

### Morphological/physicochemical and electrochemical characterization of the material

Surface morphologies of the samples were analyzed by FESEM. The EDX analysis was employed to determine the surface elemental composition of as-synthesized nanohybrid aerogel. Moreover, the structural properties of the samples were analyzed via FTIR and XRD. Thermal analysis of nanomaterial was performed in a nitrogen gas environment by scanning a temperature of 10 °C /min in a temperature range of 50–1000 °C with a Thermogravimetric and Differential Thermal Analyzer (TG/DTA).

The electrochemical performance of the as-assembled supercapacitor cell was assessed by calculating the specific capacitance (*C*; F/g), energy density (*E*; Wh/kg), and power density (*P;* W/kg), and capacitance retention values (for detailed information see S.M.)^30,31^.

## Results and discussion

### Physicochemical characterization of MWCNT/Ruthenium hydroxide aerogel

**Figure 1:**
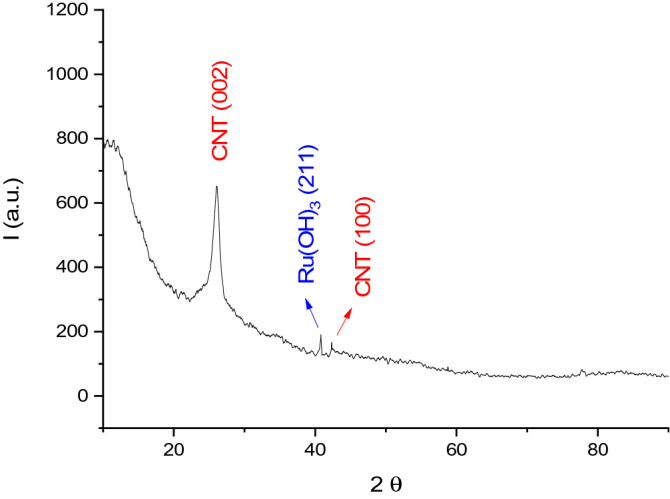
XRD diffraction patterns of MWCNT/Ruthenium hydroxide aerogel between 2θ = 10°–90°

Figure 1 depicts the X-ray diffraction patterns of MWCNT/Ruthenium hydroxide aerogel. The strong peak at about 26° belongs to CNT, and it is also a hexagonal graphite-structured peak with (002) index. At approximately 42°, a peak is observed with (100) index belonging to CNT^32^. The weak peak of Ruthenium hydroxide at about 40° is indexed by the (211) orientation^33^. Here, some diffraction peaks of Ruthenium hydroxide and MWCNT are either too weak or not visible, which is due to the very low amount of Ruthenium hydroxide in the structure and the fact that CNT is a good X-ray absorber material.

**Figure 2:**
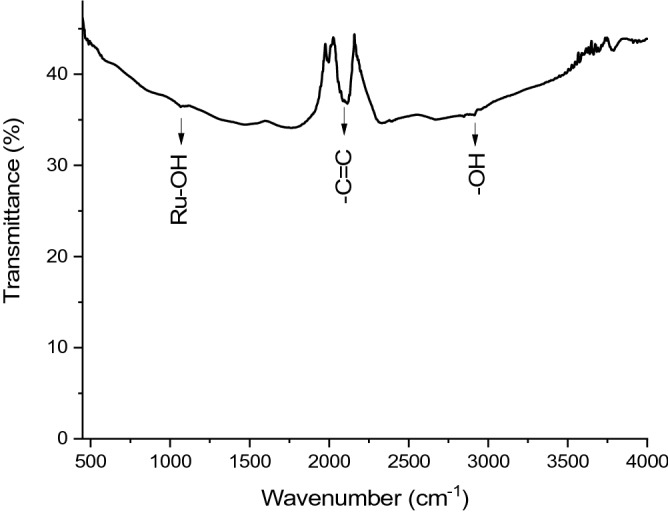
FTIR analysis and vibration peaks of MWCNT/Ruthenium hydroxide aerogel.

Figure 2 shows FTIR spectra of MWCNT/Ruthenium hydroxide nanohybrid collected in the wavelength range of 450–4000 cm^−1^ depicting the chemical bondings of aerogel (Fig. 2). The absorption band of Ru(OH)_3_ at around 1067 cm^−1^ is assigned to the characteristic stress vibrations of the Ru-OH bond. The peak at around 2928 cm^−1^ shows the –OH (bending and stretching) vibrations of Ru-OH. The peak belonging to stretching stress vibrations –C = C double bond is clearly apparent^34–36^. The peaks of the vibration bands seen in the IR analysis are much less than expected because the symmetrical structure of MWCNT causes the following. (1) The emergence of distorted bands corresponding to the absorption of the same frequency and the convergence of these vibrations' bands having very close frequencies (2) The failure to achieve the required change of dipole moment due to the symmetry of the bonds between carbon atoms.

**Figure 3:**
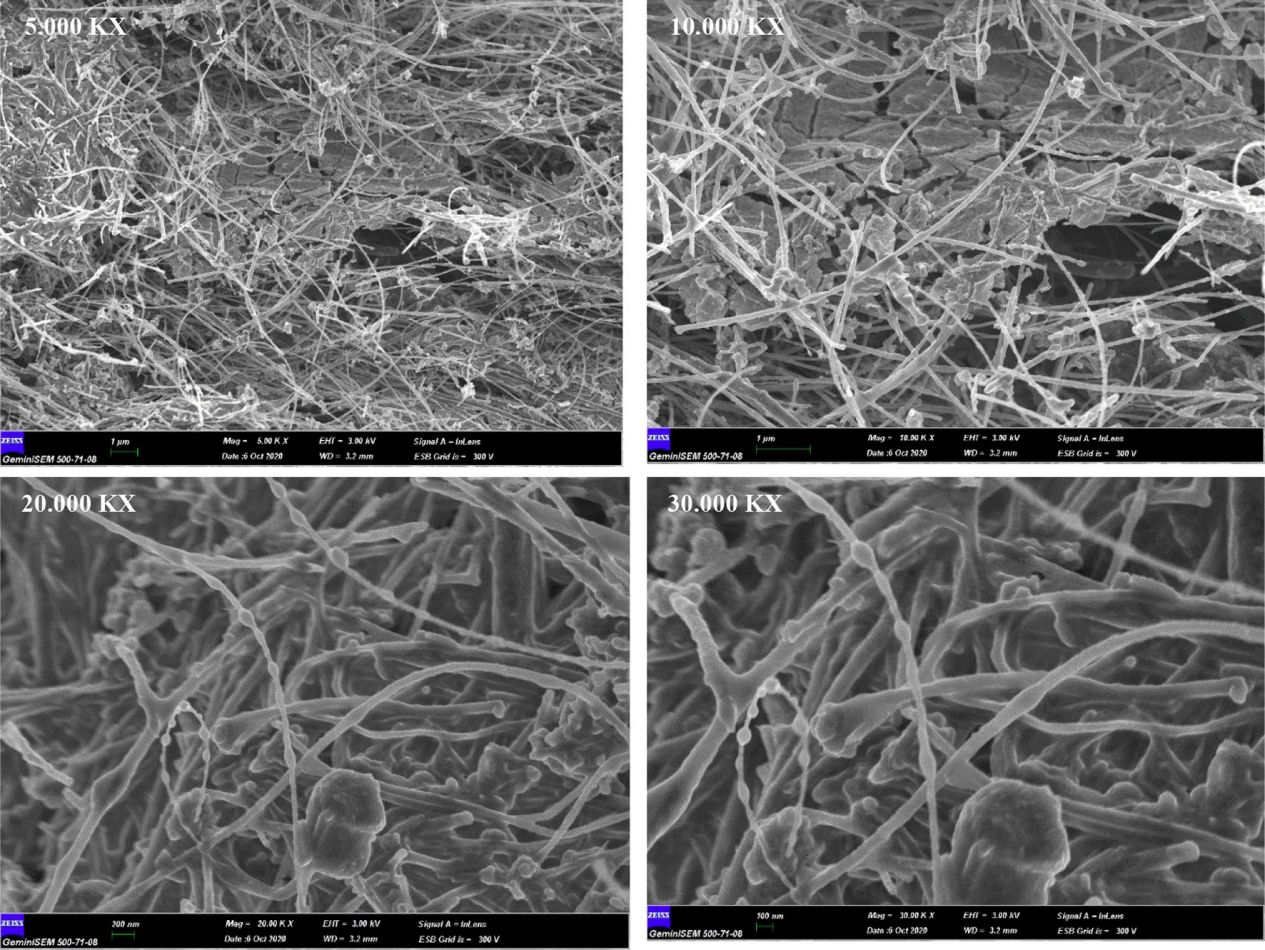
SEM images of MWCNT/Ruthenium hydroxide aerogel.

Structural characterization and elemental composition of MWCNT/Ruthenium hydroxide nanohybrid have been evaluated via SEM analysis (Fig. 3). It has been observed that RuO2 nanoparticles have been uniformly-dispersed onto the MWCNT surface. Moreover, it is worth mentioning that although the MWCNTs have been densely packed, there are still some vacancies between the carbon nanotubes. Hence, it is expected to provide a porous structure along with a high specific surface area, thereby enhancing the electrochemical performance of the electrodes. The elemental composition of MWCNT/Ruthenium hydroxide nanohybrid acquired by EDX analysis has been tabulated in Table 1. This result is consistent with XRD and FTIR data. 2.97% Ru has been detected in the structure, explaining why some peaks are not visible in both FTIR and XRD spectra.

**Table 1:** EDX analysis results of MWCNT/Ruthenium hydroxide electrodes.

Element	Weight %	Atomic %
C	37.25	51.12
O	44.56	45.91
Ru	18.2	2.97

**Figure 4:**
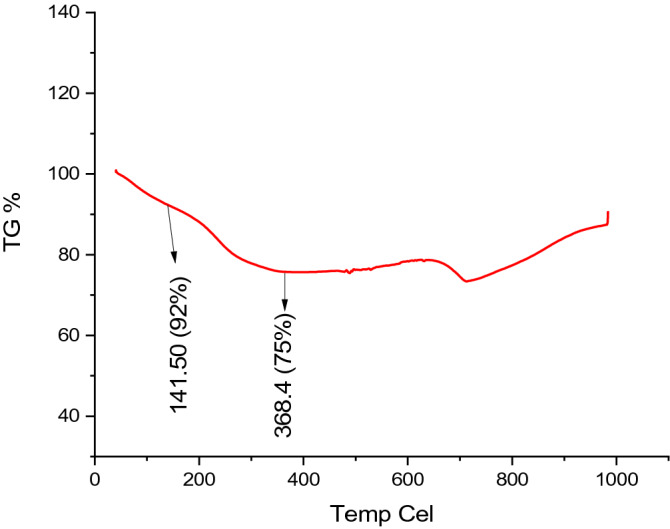
TGA curve of MWCNT/Ruthenium hydroxide aerogel.

TGA curve of as-fabricated nanomaterial was provided in Fig. 4. According to the results of the analysis, it was revealed that there was a low amount of ruptures caused by the dehydration of water molecules in the structure at 141.50 °C. Meanwhile, it was determined that water was completely removed from the material at 368.4 °C. As stated in the production method, water constituted approximately 75 wt.% of the structure. The decomposition ruptures of MWCNT were detected at 600–700 °C in accordance with the literature^37^.

### Evaluation of the electroanalytical performance of symmettric supercapacitor

**Figure 5:**
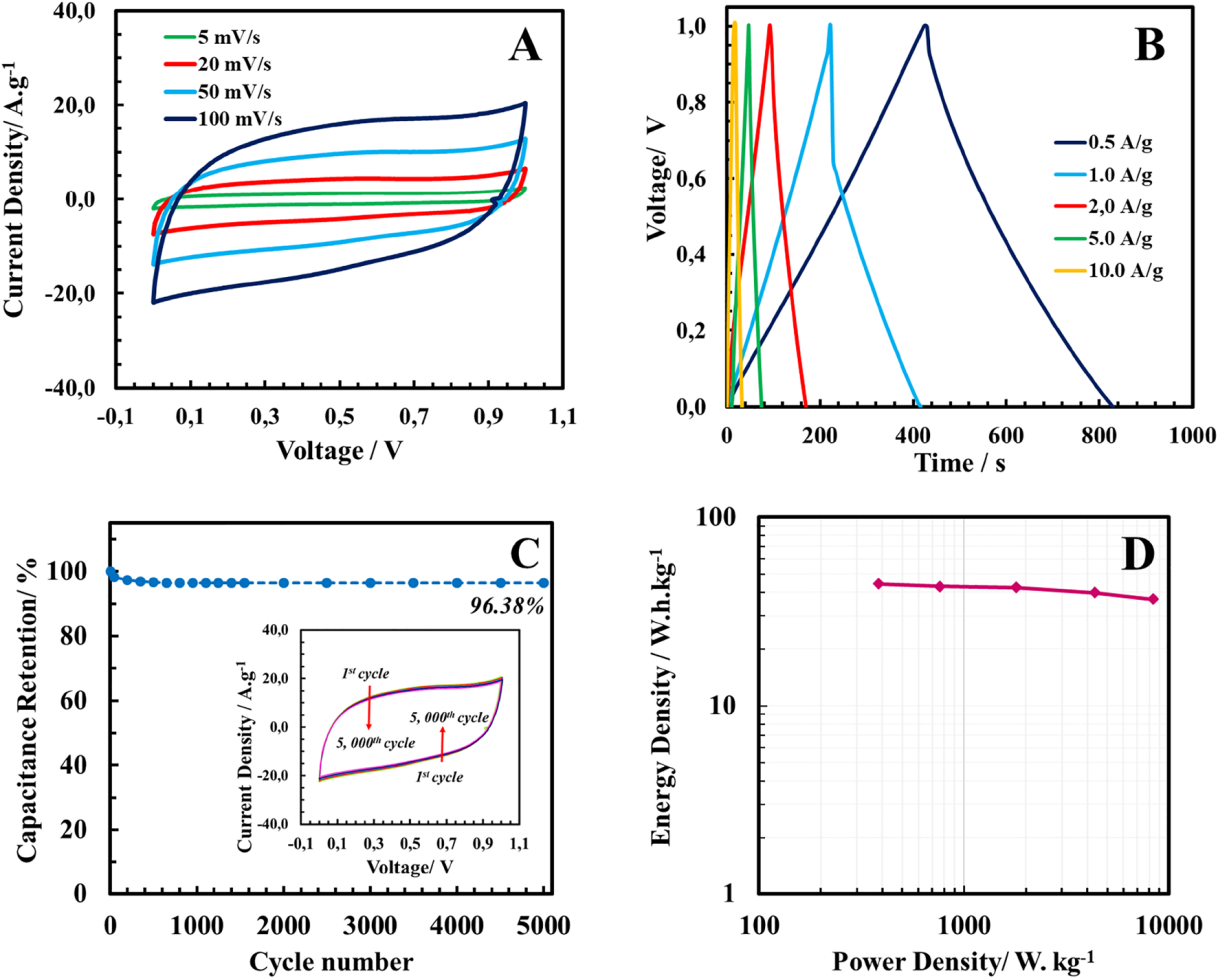
(**A**) Cyclic voltammograms of MWCNT/Ruthenium hydroxide aerogel at various potential scan rates (**B**) Galvonastatic charge/discharge curves at different current densities (**C**) Capacitance retention of MWCNT/Ruthenium hydroxide aerogel for 5000 CV cycles at 100 V.s^−**1**^ (**D**) Ragone plot of symmetric supercapacitor based on electrode active material mass.

The electroanalytical performance of the MWCNT/Ruthenium hydroxide electrode-based supercapacitor has been assessed by cyclic voltammetry and galvanostatic charge/discharge analysis. The CV curves of the supercapacitor (Fig. 5A) have offered a semi-rectangular shape indicating the synchronous effect of the pseudocapacitive behavior of the Ruthenium hydroxide nanoparticles and the electrical double layer capacitive behavior of MWCNT. Thanks to the porous structure connected to each other throughout the three-dimensional (3D) architecture, the mass transfer limitations caused by the migration of electrolyte ions are decreased; thereby, the achieved current density is high. There are no obvious anodic/cathodic peaks, which may be explained by the fast, bidirectional redox reactions that occur on the surface of fine Ruthenium hydroxide nanoparticles. Moreover, even at a higher potential scan rate of 100 mV s^−1^, the MWCNT/Ruthenium hydroxide aerogels with 3D porous structure exhibit almost ideal reversible capacitive activity, revealing outstanding capacitive performance. At a potential scan rate of 5 mV s^−1^, the maximum specific capacitance of MWCNT/Ruthenium hydroxide aerogel bases supercapacitor cell has been calculated as *ca.*423 F.g^−1^.

GCD curves of as-prepared MWCNT/Ruthenium hydroxide aerogels exhibit an almost symmetrical triangular shape, suggesting its excellent capacitive features (Fig. 5B). The calculated specific capacitance values from GCD curves are consistent with GCD curves (Table 2.). *C*_*GCD*_ values of the as-synthesized sample are 420.3 F.g^−1^ and 319.9 F.g^−1^ at current densities of 0.5 A.g^−1^ and 10.0 A.g^−1^. The specific capacitance values of MWCNT/Ruthenium hydroxide aerogels calculated from galvanostatic charge/discharge curves are relatively higher than the reported values for similar nanocomposites. This outstanding electrochemical performance is ascribed to the 3D-ordered electrochemically accessible surface area and the metal oxide decoration on the MWCNT surface.

**Table 2:** Electroanalytical performance metrics of MWCNT/Ruthenium hydroxide aerogel-based supercapacitor.

Current density (A.g^−1^)	Specific capacitance (F. g^−1^)	E (W.h.kg^−1^)	P (W. kg^−1^)
0.5	420.3	44.4	383.3
1.0	406.3	42.9	762.3
2.0	369.1	42.3	1797.6
5.0	347.4	39.9	4347.4
10.0	320.0	36.7	8363.7

Moreover, 5000 consecutive CV cycles at a potential scan rate of 100 mV s^−1^ were conducted to evaluate the sample's cyclic stability (Fig. 5C). As can be observed from the inset of Fig. 5C, comparing the 5000th and the 1st cycle, the unaltered shape of CV voltammograms reveals the high cyclic stability of the aerogel. As described in S.M., the capacitance retention values have been computed by the specific capacitance values calculated from CV curves. The capacitance retention of the as-assembled supercapacitor is found to be 96.38% for the 5,000 CV cycle (Fig. 5C).

The Ragone plot of MWCNT/Ruthenium hydroxide aerogel-based supercapacitor has been depicted in Fig. 5D. Table 2 summarizes the computed energy density and power density values. The energy density of MWCNT/Ruthenium hydroxide aerogel-based supercapacitor has been calculated as high as 36.6 Wh kg^−1^ even at a high power density value of 8.60 kW kg^−1^. Moreover, the slope of the Ragone diagram tends to become linear even at higher power density values, implying the superior electrochemical characteristics of the supercapacitor cell. These findings reveal that thanks to its comparable energy density values with commercially available Pb-acid or Ni-metal hybrid energy storage systems, the fabricated supercapacitor cell is promising to be utilized as an alternative high-performance energy storage system.

In Table 3, the examples of carbon nanotube ruthenium supercapacitors, which are well known in the literature, are displayed. Accordingly, in studies where a higher capacitance than this study was reported, either Pd thin films were produced as the substrate, or very strong electrolytes (consisting of metal salts) were used. There is no other study like this one, with high capacitance and 96.3% retention rate in 5000 cycles.

**Table 3:** Comparison of the Study with the Literature.

Study	Method	Scan rate (mV/s)	Capacitance (F/g)	Cycle/retention
^23^	Hydrothermal	2	1585	20,000 cycle/88%
^24^	Porous film	50	1084	300 cycle/N/A
^25^	Thick film	20	176	10,000 cycle/97%
^26^	Hydrothermal	100	80	100 cycle/N/A
This study	Aerogel	5	420	5000 cycle/96.3%

## Conclusion

Herein, a facile and straightforward production pathway has been proposed for the fabrication of high-performance supercapacitor cells. In this regard, MWCNT/Ruthenium hydroxide aerogel has been synthesized and utilized as an electrode active material for a symmetrical supercapacitor. The specific capacitance values have been calculated to be 420.3 F.g^−1^ and 319.9 F g^−1^ at the current density of 0.5 A g^−1^ and 10.0 A g^−1^, respectively. The cyclic stability of the supercapacitor has been assessed for 5000 consecutive CV cycles, and the capacitance retention has been found to be 96.38%. Moreover, the assembled MWCNT/Ruthenium hydroxide aerogel-based supercapacitor cell has offered a superior energy density of 36.6 Wh kg^−1^ even at a high power density of 8.36 kW kg^−1^. The findings confirm that as-synthesized MWCNT/Ruthenium hydroxide aerogel can be successfully utilized as a high-energy supercapacitor electrode material. Thus, it can be speculated that this work paws the way for engineering and designing high-performance energy storage systems based on hybrid nanomaterials.

## Supplementary Information

Supplementary Information.

## Data Availability

The datasets supporting the conclusions of this article are included within the article.
