# Supplementary material for: MWCNT/Ruthenium hydroxide aerogel supercapacitor production and investigation of electrochemical performances
Source: Sci Rep. 2022 Jul 27;12:12862. doi: 10.1038/s41598-022-17286-w (PMC9329456; doi:10.1038/s41598-022-17286-w)
Supplement: Supplementary file 1 — Supplementary Information. [file 41598_2022_17286_MOESM1_ESM.docx]

**Supplementary Materials for**

MWCNT/ Ruthenium hydroxide **AEROGEL SUPERCAPACITOR PRODUCTION AND INVESTIGATION OF ELECTROCHEMICAL PERFORMANCES**

**Satiye Korkmaz^a^, İ. Afşin Kariper^b*^ Ceren Karaman^c^, Onur Karaman^d^**

^a^Karabuk University, Faculty of Engineering, Department of Electrical-Electronics Engineering, 78050, Karabük, Turkey

^b^Erciyes University, Education Faculty, 38039, Kayseri, Turkey

^c^Akdeniz University, Department of Electricity and Energy, 07058, Antalya, Turkey

^d^Akdeniz University, Department of Medical Imaging Techniques, 07058, Antalya, Turkey

***Evaluation of Electrochemical Performance of Supercapacitor Cells:***

The total specific capacitance, C*_cv_*(F/g) of the symmetric supercapacitor cells was computed from the CV curves by using the Eq. 1;

$C_{cv}=\frac{1}{V*v}\int_{V_{-}}^{V_{+}} i(V)dV=\frac{q_{a}+\left| q_{c} \right|}{m. \Delta V}$ (1)

where *q_a_*, *q_c_*, and *m* are the anodic and cathodic voltammetric charges on positive and negative sweeps, m is the total mass of active material, respectively. *i(V)* is the current in a CV curve, *v* is the potential scan rate, and *∆V* is the voltage range of cyclic voltammetry (V) [1-2]

The specific capacitance *C_GCD_*(F/g) of the symmetrical supercapacitor cell was calculated from the GCD curve by Eq.2:

$C_{GCD}=\frac{2 I t_{d}}{m \Delta V}$ (2)

where *I* is the constant current (A), *m* is the mass of active material (g) in one electrode, *t_d_* is the discharge time, and ∆*V* is the potential range excluding the voltage drop (*V_drop_*) at the beginning of the discharge.

The energy density, *E*(Wh/kg), was calculated using the Eq. (3);

$E=\frac{1}{7.2}C_{GCD}{\Delta V}^{2}$ (3)

The power density, *P*(W/kg), was estimated using the Eq. (4);

$P=\frac{E}{t_{d}}$ (4)

where *t_d_* (h) is the discharge time at a constant current density

The capacitance retention was calculated according to Eq. (5);

$Capacitance Retention \left( \% \right)=\frac{C_{i}}{C_{1}}\times100$ (5)

C_i_ and C_1_ are the specific capacitances at i^th^ and 1^st^ CV cycles [1-2].

**References**

**[1]** Subramanya, B., & Bhat, D. K. (2015). Novel eco-friendly synthesis of graphene directly from graphite using 2, 2, 6, 6-tetramethylpiperidine 1-oxyl and study of its electrochemical properties. *Journal of Power Sources*, *275*, 90-98.

**[2]** Wang, H., Yi, H., Chen, X., & Wang, X. (2014). Asymmetric supercapacitors based on nano-architectured nickel oxide/graphene foam and hierarchical porous nitrogen-doped carbon nanotubes with ultrahigh-rate performance. *Journal of Materials Chemistry A*, *2*(9), 3223-3230.
